# Multi-target drug repositioning by bipartite block-wise sparse multi-task learning

**DOI:** 10.1186/s12918-018-0569-7

**Published:** 2018-04-24

**Authors:** Limin Li, Xiao He, Karsten Borgwardt

**Affiliations:** 1 School of Mathematics and Statistics, Xi’an Jiaotong University, Xi’an, 710049 China; 2 Department of Biosystems Science and Engineering, ETH Zurich, Basel, Switzerland; 3 Swiss Institute of Bioinformatics (SIB), Basel, Switzerland

**Keywords:** Drug repositioning, Multi-task learning, L1000

## Abstract

**Background:**

Finding potential drug targets is a crucial step in drug discovery and development. Recently, resources such as the Library of Integrated Network-Based Cellular Signatures (LINCS) L1000 database provide gene expression profiles induced by various chemical and genetic perturbations and thereby make it possible to analyze the relationship between compounds and gene targets at a genome-wide scale. Current approaches for comparing the expression profiles are based on pairwise connectivity mapping analysis. However, this method makes the simple assumption that the effect of a drug treatment is similar to knocking down its single target gene. Since many compounds can bind multiple targets, the pairwise mapping ignores the combined effects of multiple targets, and therefore fails to detect many potential targets of the compounds.

**Results:**

We propose an algorithm to find sets of gene knock-downs that induce gene expression changes similar to a drug treatment. Assuming that the effects of gene knock-downs are additive, we propose a novel bipartite block-wise sparse multi-task learning model with super-graph structure (BBSS-MTL) for multi-target drug repositioning that overcomes the restrictive assumptions of connectivity mapping analysis.

**Conclusions:**

The proposed method BBSS-MTL is more accurate for predicting potential drug targets than the simple pairwise connectivity mapping analysis on five datasets generated from different cancer cell lines.

**Availability:**

The code can be obtained at http://gr.xjtu.edu.cn/web/liminli/codes.

## Background

In recent years, *multi-target* drugs - that is, drugs that affect more than one gene or protein - have been moving into the focus of drug discovery and development [1, 2]. The first reason for this phenomenon is that multi-target drugs have been found to be more effective than single-target alternatives for several complex diseases, such as cancer and metabolic diseases [1, 3–5]. The rationale behind this observation is that the efficacy of the inhibition of a single target may often not be strong enough to affect the entire biological process, which means that multiple targets with weaker inhibition may have a stronger combined effect than a single blocked target. A second reason to study multi-target drugs is that many drugs fail to be approved because of their severe side effects in clinical trials [2, 6], which is a negative consequence of more than one target being affected. Therefore, finding potential compound targets is a crucial step in *drug profiling*, the process that seeks those compounds with a desired target or those without undesired side effects.

Many machine learning methods have been proposed for finding potential drug targets based on compound structure [5]. The rationale is that if two compounds are similar in structure, they may have similar targets. The targets of the compounds are inferred by comparing their structures to known drugs. However, it has been shown that many compounds with similar structure have different effects [7]. Therefore, considering only structure information is not sufficient to accurately detect potential drug targets. Several other types of information are also used for drug target prediction, such as drug sensitivity, drug side effects, gene expression, gene/protein structure, gene/protein function, Protein Protein Interaction (PPI) or metabolic network [8–12]. In recent work, Liu et al. [13] sought to solve the drug targeting problem by using a new type of information in form of the LINCS L1000 dataset [14], which includes expressions levels of single gene knock-downs and drug treatments. This information connect drugs and gene knock-downs directly through their regulation effects on all the genes in a cell.

This LINCS L1000 dataset [14] is a part of the Library of Integrated Network-Based Cellular Signatures (LINCS) Program (http://www.lincsproject.org/) that generates and publishes large datasets of measurements that quantify how cells respond to a variety of perturbing agents. Specifically, the LINCS L1000 platform (http://www.lincscloud.org/) provides large-scale gene expression assays in which cultured cells have been exposed to various chemical and genetic perturbations [14]. The LINCS L1000 dataset includes 20,413 small-molecule compounds and 18,493 shRNAs knock-downs tested in 18 different cancer cell lines. After each perturbation, a gene expression profile for each cell line is obtained. This huge dataset creates the opportunity to analyze the relationship between compounds and gene targets at a genome-wide level.

Liu et al. [13] explored this relationship based on the assumption that a drug treatment and the knock-down of a target gene of this drug will induce similar gene expression changes in a sample. Using this idea, drug targets can be inferred by *connectivity mapping analysis* [15], that is, by finding knock-downs and drugs with similar gene expression profiles. Similarity between gene expression profiles is determined using the gene set enrichment analysis [16] that quantifies whether a drug and a gene knock-down up- or down-regulate the same set of genes.

Connectivity mapping-based approaches [13, 15] lead to a one-to-one mapping between drugs and gene knock-downs. However, the effect of a drug may not resemble that of only knocking down its single-target gene. Many drugs are able to inhibit several known target genes and many closely related genes on various biology pathways. If a drug inhibits many genes, the gene expression measured after the drug treatment may be different from those measured after each of the gene knock-down experiments. Connectivity mapping ignores the additive effects of gene knock-downs which exist in many biological systems [17–19].

Therefore, our goal in this paper is to develop an approach for multi-target drug repositioning using the LINCS L1000 dataset that could overcome the restrictive assumptions of connectivity analysis. We model the problem as finding combinations of gene knock-downs that induce gene expression changes similar to a drug treatment. Furthermore, we assume that the effect of a drug treatment can be modelled as the additive effects of all its single target gene knock-downs, which is reasonable since additive effects of gene knock-downs exist in many biological systems [17–19]. Finally, we propose an efficient and effective multi-task machine learning approach for detecting the potential drug targets, using both expression data and compound structure information. The assumption of additive effects of gene knock-downs may not reveal the true underlying biology system. However, our experiments show that, in a practical sense, it works much better than pairwise connectivity mapping in predicting the potential drug targets.

The analysis of the LINCS L1000 dataset is further complicated by the fact that each drug treatment is replicated in several plates, each of which represents one gene expression *signature* of the drug treatment. This is similar for the gene knock-down, where each genetic perturbation is performed as a knock-down of one of the shRNAs of the gene. Therefore, each gene knock-down is represented by several signatures as well, which may vary for different shRNAs. These replication experiments make the data set more reliable, as the redundancy in measurements will lead to noise reduction and to a better representation of the spectrum of the effects of a drug. However, this also makes the data analysis more complicated. For example, the enrichment analysis-based methods cannot be directly applied to test the association between drugs and gene knock-downs, since drug treatments and gene knock-downs are represented by set of signatures.

We propose a novel bipartite block-wise sparse multi-task learning method that detects the relationships between groups of drug signatures and groups of gene signatures in an unsupervised manner. The optimization problem can be solved based on the accelerated proximal gradient method, which is more efficient than the computationally demanding enrichment analysis-based test. In terms of effectiveness, our extensive experiments on five cancer cell lines from the LINCS L1000 data [14] provided more accurate predictions of potential drug targets than the simple connectivity mapping-based test, validated by known drug targets from the DrugBank database [20], together with the Gene ontology (GO) function information from the GO database [21], or with PPI information from the HPRD PPI network [22]. The prediction results generate an interesting unified connected bipartite graph of drugs and genes across different cell lines, where we can find co-modules of drugs and genes, duplicate edges across multiple cell lines, and meaningful connections of genes in the same pathways. These novel and meaningful discoveries from the L1000 database demonstrate the effectiveness of our approach.

## Methods

### Materials

We downloaded the small-molecule compound and shRNA data in the L1000 dataset, which was released by the Broad Institute LINCS Data Generation Center [14]. In our experiments, we used the expressions for the 978 landmark genes in the five cell lines of SW480, HT29, HCC515, MCF7, and PC3. A landmark gene is one whose gene expression has been determined as being informative to characterize the transcriptome and which is measured directly in the L1000 assay. The 978 landmark genes were selected as those widely expressed across lineage and were found to have good predictive power for inferring the expression of other genes that are not directly measured in the assay. For each of these cell lines, we generated a smaller dataset, which included only the treatment effects for the approved drugs and shRNA genes in the DrugBank Database [20]. For the drug treatments or the gene knock-downs in each dataset, the same treatment conditions were used. For all the cell lines except SW480, the drug treatment with dose of 10*μm* and duration of 24*h* was used. For the SW480, there are no experiments for drug treatments with duration 24*h*, so we used the duration of 6*h* instead. The details of the data information are shown in Table 1.

**Table 1:** Data information for the five datasets

Cell line	No.drugs	No.d-treats	D-dose	D-time	No. genes	No.g-treats	G-time
HCC515	144	504	10 *μm*	24*h*	156	1715	96*h*
HT29	44	160	10 *μm*	24*h*	174	2543	96*h*
PC3	329	2513	10 *μm*	24*h*	223	2954	96*h*
SW480	4	8	10 *μm*	6*h*	6	36	96*h*
MCF7	293	1608	10 *μm*	24*h*	219	2655	96*h*

We also downloaded the drug structure from the KEGG database [23] and computed the structure similarities among the drugs by applying the software Simcomp [24] on the drug structures.

### Approach

**Fig. 1:**
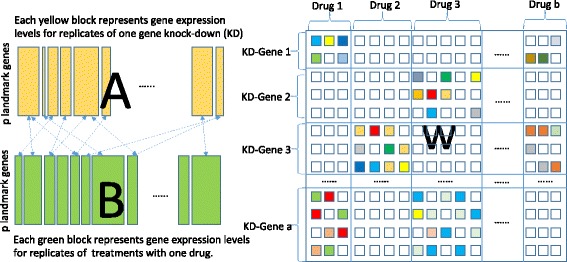
Illustration of the proposed model. Each block of *A* and *B* represents differential gene expression data after gene knock-down and drug treatment. *W* is the association matrix we would like to learn

Suppose we have tested the responses of a cell line after *b* treatments with small-molecule compounds and *a* gene knock-downs. After the treatments, the expression levels of *p* selected landmark genes are evaluated. As there are several replicates of each perturbation, there are several signatures for each drug treatment or gene knock-down treatment. For gene knock-downs we obtain a *p*×*m* differential gene expression matrix *A*, where *m* is the total number of experiments with a gene knock-down. The effect of each gene therapy *i*∈{1,⋯,*a*} is represented by a column block of *A*, as demonstrated in Fig. 1, with *m*_*i*_ replicating experiments, where $\sum _{i} m_{i}=m$. Similarly, we get another *p*×*n* differential gene expression matrix *B* for the drug treatment. The effect of each drug therapy *j*∈{1,⋯,*b*} is represented by a column block of *B* with *n*_*j*_ replicating experiments, where $\sum _{j} n_{j}=n$. For the sake of simplicity, we have assumed the columns with the same gene knock-down in *A* or the same drug in *B* are grouped together. Table 2 summarizes the notations used in this paper.

**Table 2:** List of notations

Notation	Description
*A*	*p*×*m* matrix with gene expressions for gene therapies.
*B*	*p*×*n* matrix with gene expressions for drug treatments.
*K*	*b*×*b* similarity matrix among *b* drugs.
*p*	number of landmark genes whose gene expressions are measured.
*a*	number of knockout genes, or the number of column blocks in A.
*b*	number of drugs, or the number of column blocks in B.
*m*_*i*_	number of signatures for knocking down gene *i*.
*n*_*j*_	number of signatures for treatments using drug *j*.
*m*	$\sum _{i} m_{i}$,total number of experiments with gene knockout.
*n*	$\sum _{j} n_{j}$,total number of experiments with drug treatment.
*W*	*m*×*n* matrix with multivariate factors
*W* ^*ij*^	*m*_*i*_×*n*_*j*_ matrix, the (*i,j*) block in *W*
*W* ^:*j*^	*m*×*n*_*j*_ matrix, the (·,*j*) sub-matrix in *W*
*W* ^*i*:^	*m*_*i*_×*n* matrix, the (*i*,·) sub-matrix in *W*
*K*(*s,t*)	*k*_*st*_, the (*s,t*) entry in K.
$w^{j}_{l}$	the *l*th column in the block *W*^:*j*^
$\bar {w}_{j}$	mean of the columns in *W*^:*j*^

As mentioned, the aim of this paper is to find sets of gene knock-downs that could induce gene expression changes similar to those of a drug treatment. In other words, we would like to learn a weight matrix *W* (see Fig. 1), such that each block of *B* can be approximated by a combination of blocks in *A*.

Therefore, our objective function can be represented as:

1 $$ \begin{array}{l} \Omega(W)=L(A,B,W) + \Phi(W), \\ \end{array}  $$

where *L*(*A,B,W*) is a loss function and *Φ*(*W*) is a regularization term. This is a typical multi-task learning problem [25], where a *task* corresponds to a column of *W* and a *feature* corresponds to a row of *W*.

In our biological application, a task represents a specific signature for a drug treatment and a feature represents a specific signature for a gene knock-down. Thus, each drug treatment with multiple signatures corresponds to a group of tasks, and each gene knock-down with multiple signatures corresponds to a group of features.

As we consider a multivariate regression model, the loss function can be represented as $L(A,B,W) =\frac {1}{2} \|B-AW\|_{F}^{2}.$ Since a drug often affects a few genes [20], we would like *W* to have a sparsity structure. Two common regularization terms for sparsity are

$$ \begin{array}{l} \Phi_{1}(W)=\|W\|_{1} = \sum_{k=1}^{m} \sum_{l=1}^{n}|W(k,l)|, \end{array} $$

which enforces sparse entries in *W*, and

$$ \begin{array}{l} \Phi_{2,1}(W)=\|W\|_{2,1} = \sum_{k=1}^{m}\|W(k,:)\|_{2}, \end{array} $$

which enforces row sparsity in *W*. Here, *W*(*k,l*) and *W*(*k*,:) represent the (*k,l*) entry and the *k*-th row of *W*, respectively. The *ℓ*_1_ and *ℓ*_2,1_ regularizations are widely used in multi-task learning approaches in various applications, including bioinformatics [25–28]. *ℓ*_2,1_ sparsity is usually called group sparsity or block sparsity in multi-task learning since it is generalized from group lasso and assumes that only a few features should be selected for all the tasks.

However, in our scenario we do not want sparse entries in *W* with *ℓ*_1_ regularization or sparse rows in *W* with *ℓ*_2,1_ group sparsity regularization. As demonstrated in Fig. 1, we would like *W* to be sparse block-wise in a bipartite way, such that a few groups of features are selected for a group of tasks. As a result, each drug only has a small number of potential targets.

In “Bipartite block-wise sparse multi-task learning” section, we propose a bipartite block-wise sparse multi-task learning model and an efficient optimization algorithm to solve the problem. In “Graph structure on group of tasks” section we integrate the compound structure information into the proposed bipartite block-wise sparse multi-task learning model. In “Association stability score” section we introduce the stability selection strategy for parameters.

#### Bipartite block-wise sparse multi-task learning

In this section, we propose a new type of sparsity called *bipartite block sparsity* that enforces sparse blocks in the *W* matrix such that a few groups of features are selected for each group of tasks. Suppose $W^{ij}\in \mathbb {R}^{m_{i}\times n_{j}} $ represents the (*i,j*) block in *W* corresponding to the *i*-th gene and *j*-th drug. Our bipartite block sparsity (*ℓ*_*bb*_) regularization is represented as

$$ \begin{array}{l} \Phi_{bb}(W)=\sum_{i=1}^{a}\sum_{j=1}^{b}\left\|W^{ij}\right\|_{F} \end{array} $$

With slightly changed *ℓ*_1_ regularization and our *ℓ*_*bb*_ regularization together, we proposed a bipartite block-wise sparse multi-task model as

2 $$ \begin{array}{l} \min\limits_{W} \frac{1}{2}\| B-AW\|_{F}^{2} + \sum\limits_{i,j}^{a,b} \alpha_{ij}(\lambda_{1} \left\|W^{ij}\right\|_{1} + \lambda_{2} \left\|W^{ij}\right\|_{F})  \end{array}  $$

where *α*_*ij*_ is a weight factor for each block and can be simply chosen as number of entries in the (*i,j*) block in *W*. Note that when *α*_*ij*_=1, the first regularization term is equal to the *ℓ*_1_ regularization.

#### Graph structure on group of tasks

The chemical structure of the drugs is commonly used in drug target discovery, since similar drugs often share similar targets [5]. Suppose we are given a matrix of $K\in \mathbb {R}^{b\times b}$ that contains structure similarity among all the *b* drugs in our scenario. *K* can be considered as a graph matrix or adjacent matrix of a graph. To integrate drug structure information in the above model (2), we further develop a novel multi-task learning model with a *super-graph* structure.

In general multi-task learning, graph structure on tasks may increase the accuracy for multi-task learning [28]. Instead of graph structure on tasks, our application should consider the graph structure on groups of tasks, which we refer to as *super-graph* structure.

Suppose the *j*_1_-th task group (*j*_1_-th drug) has a weight score of $k_{j_{1}j_{2}}=K(j_{1},j_{2})$ with the *j*_2_-th task group (*j*_2_-th drug). Denote the submatrix in *W* corresponding to the *j*-th drug by *W*^:*j*^, the *l*th column in the matrix *W*^:*j*^ by $w^{j}_{l}$, and the mean of the columns in *W*^:*j*^ by $\bar {w}_{j}$. The super-graph structure can be used for another type of regularization:

3 $$ \begin{array}{l} \Phi_{s}(W)= \sum\limits_{j=1}^{b}n_{j}\sum\limits_{l=1}^{n_{j}}\left\|w^{j}_{l}-\bar{w_{j}}\right\|_{2}^{2}+ \sum\limits_{j_{1}j_{2}}\left\|\bar{w}_{j_{1}}-\bar{w}_{j_{2}}\right\|_{2}^{2}k_{j_{1}j_{2}}. \end{array}  $$

The first term ensures that the tasks in each group are as close to the group center as possible, and the second term ensures that the group centers have the graph structure that is represented by *K*.

Putting all these components together, we propose the bipartite block-wise sparse multi-task learning model with super-graph structure (BBSS-MTL)

4 $$ \begin{array}{ll} \min\limits_{W} \Omega(W) &= \min\limits_{W} \frac{1}{2}\left\| B-AW\right\|_{F}^{2} + \sum\limits_{i,j}\alpha_{ij}\left(\lambda_{1}\left\|W^{ij}\right\|_{F}\right.\\ &\quad\left.+\lambda_{2}\left\|W^{ij}\right\|_{1}\right)+\lambda_{3} \Phi_{s}(W).\\ \end{array}  $$

### Optimization algorithm

To solve this optimization problem, we first simplify the terms in (3). The first term in (3) can be rewritten as

5 $$ \sum\limits_{j=1}^{b}n_{j}\sum\limits_{l=1}^{n_{j}}\left\|w^{j}_{l}-\bar{w_{j}}\right\|_{2}^{2} \,=\, \sum\limits_{j=1}^{b} \left\|\sqrt{n_{j}}W^{:j}H_{j}\right\|_{F}^{2} =\text{tr}\left(\!W\tilde{H}W^{T}\!\right),   $$

where $H_{j}=I_{n_{j}}-e_{n_{j}}e_{n_{j}}^{T}/n_{j}$ is a centering matrix, $e_{n_{j}}$ is an *n*_*j*_-dimensional column vector with all ones, and $\tilde {H} = \text {diag}\left (n_{1}H_{1},\cdots,n_{b}H_{b}\right)$. Suppose the Laplacian matrix of the graph is *L*=*D*−*K*, where *D*=diag(*d*_1_,⋯,*d*_*b*_) is a diagonal matrix with $d_{j} = \sum _{k} K(j,k)$. The second term in (3) can be simplified as $tr(\bar {W}L\bar {W}^{T})$, where $\bar {W} = [\bar {w}_{1},\cdots,\bar {w}_{b}]$. Note that $\bar {W}$ can be further simplified as

$$\begin{aligned} \bar{W} \,=\, \left[\!\frac{W^{:1}e_{n_{1}}}{n_{1}},\cdots\!,\frac{W^{:b}e_{n_{b}}}{n_{b}}\!\right] = \left[\!W^{:1},\cdots,W^{:b}\!\right]\text{diag}\left(\!\frac{e_{n_{1}}}{n_{1}},\cdots\!,\frac{e_{n_{b}}}{n_{b}}\!\right) \!=WE, \end{aligned} $$

where $E=\text {diag}(e_{n_{1}}/n_{1},\cdots,e_{n_{b}}/n_{b})$ is a block diagonal matrix. Thus the second term in (3) can be simplified as

6 $$ \begin{array}{l} \text{tr}\left(WELE^{T}W^{T}\right).\\ \end{array}  $$

From (5) and (6), we can obtain that the equation in (3) is equivalent to

7 $$ \begin{array}{l} \Phi_{s}(W) =\text{tr}\left(W\left(\tilde{H}+ELE^{T}\right)W^{T}\right) = \text{tr}\left(W\tilde{L}W^{T}\right)\\ \end{array}  $$

where $\tilde {L} = \tilde {H}+ELE^{T}$.

Thus the BBSS-MTL in model (4) can be rewritten as

8 $$ \begin{array}{l} \min\limits_{W} \frac{1}{2}\left(\!\| B-AW\|_{F}^{2} + \sum\limits_{i,j}\alpha_{ij}(\lambda_{1}\left\|W^{ij}\right\|_{F}+\! \lambda_{2}\left\|W^{ij}\right\|_{1}\!\right)\,+\, \lambda_{3} tr\left(\!W\tilde{L}W^{T}\!\right)\\ \end{array}  $$

To solve the optimization problem in Eq. 8, we propose an accelerated proximal gradient-based algorithm. The key step is to generate the proximal operator:

$$\begin{array}{lll} \text{prox}(V)& = &\text{argmin}_{W} \frac{1}{2} \|W-V\|_{F}^{2}+ \\ &&\sum_{i=1}^{a}\sum_{j=1}^{b} \alpha_{ij}\left(\lambda_{1}\|W^{ij}\|_{1} +\lambda_{2} \|W^{ij}\|_{F}\right) \\ \end{array} $$

Fortunately, this can be obtained block-by-block, as follows:

$$\begin{array}{lll} \text{prox}^{bb}(V^{ij})& = &\text{argmin}_{W^{ij}} \frac{1}{2} \left\|W^{ij}-V^{ij}\right\|_{F}^{2}+\\ &&\alpha_{ij}\left(\lambda_{1} \left\|W^{ij}\right\|_{1} + \lambda_{2}\left\|W^{ij}\right\|_{F}\right) \end{array} $$

It can be proved that the above operator exhibits a certain decomposition property, based on which we can efficiently obtain the proximal operator in two stages.

$$\text{prox}^{bb}\left(V^{ij}\right) = \text{prox}^{b2}_{\lambda_{2}}\left(\text{prox}^{b1}_{\lambda_{1}}\left(V^{ij}\right)\right),$$

where the *ℓ*_1_ proximal operator on the matrix *V*^*ij*^ is

9 $$ \begin{array}{lll} \text{prox}^{b1}_{\lambda_{1}}\left(V^{ij}\right)& =& \text{argmin}_{W^{ij}} \frac{1}{2} \left\|W^{ij}-V^{ij}\right\|_{F}^{2}+\alpha_{ij}\lambda_{1} \left\|W^{ij}\right\|_{1}\\ &=&\left(W^{ij}-\lambda_{1}\alpha_{ij}\right)_{+}-\left(-W^{ij}-\lambda_{1}\alpha_{ij}\right)_{+}\\ \end{array}  $$

and the *ℓ*_2_ proximal operator on the matrix *V*^*ij*^ is

10 $$  \begin{array}{lll} \text{prox}^{b2}_{\lambda_{2}}(V^{ij})& =& \text{argmin}_{W^{ij}} \frac{1}{2} \left\|W^{ij}-V^{ij}\right\|_{F}^{2}+\alpha_{ij}\lambda_{2} \left\|W^{ij}\right\|_{F}\\ &=&\left(1- \lambda_{2}\alpha_{ij}/\left\|W^{ij}\right\|_{F}\right)_{+}W^{ij} \\ \end{array}  $$

The pseudocode of the proposed method BBSS-MTL is shown in Algorithm 1.



### Association stability score

In this section, we provide a stability selection strategy to deal with parameters in the proposed models. Suppose we have the sets *Λ*_1_, *Λ*_2_ and *Λ*_3_ for the parameters *λ*_1_, *λ*_2_ and *λ*_3_, respectively. For each combination of the parameters *λ*={*λ*_1_,*λ*_2_,*λ*_3_}∈*Λ*_1_×*Λ*_2_×*Λ*_3_=*Λ*, we define a probability score $P_{\lambda }\in \mathbb {R}^{a\times b}$ for all blocks in *W* in the following way. We first subsample {*A*_*t*_,*B*_*t*_} from the data {*A,B*} with the number of rows being *p*/2. Using our BBSS-MTL model, we can obtain *W*_*t*_.

We repeat the procedure T times and compute the probability of hitting for each block {*i,j*} as $P_{\lambda }(i,j) = \sum _{t} \left (W_{t}^{ij}\neq 0 \right)/T$. We then compute these probability values for each combination of the parameters *λ*∈*Λ*, and define the association stability score of block {*i,j*} by averaging these probabilities over different parameters as *score*(*i,j*)=*mean*_*λ*∈*Λ*_*P*_*λ*_(*i,j*).

## Results

In this section, we evaluate our approaches on eight simulated datasets and show the effectiveness of the BBSS-MTL for bipartite block sparsity and the super-graph structure. We then apply our approach to find potential targets for drugs on datasets from five cell lines.

### Simulation

#### A. Data Generation

We simulate data using the following scenario with *p*=50,*m*=200,*n*=80,*a*=20,*b*=10. We first simulate $A\in \mathbb {R}^{p\times m}$, $W\in \mathbb {R}^{m \times n}$, and then generate $B\in \mathbb {R}^{p\times n} = AW + E$, where the elements of *E* are sampled from a standard normal distribution *N*(0,1). We assume the groups of correlated input variables in *A* have an equal size of 10.

To generate *A*, we first generate a prototype column vector $\bar {A}_{i}$ for each group *i*, where $\bar {A}_{i} \sim N(0,5I_{50})$ and *I*_50_ is a 50-dimensional identity matrix. We then generate the columns in this group by $A_{i_{k}} = \bar {A}_{i} + \epsilon $, where *ε*∼*N*(0,*I*_50_), $i_{k} \in \mathcal {I}_{i}$, $\mathcal {I}_{i}$ represents the indices in *i*-th column group of *A*. The procedure is repeated for each column group of *A*, and we get *A* with column group structure.

To generate $W\in \mathbb {R}^{m\times n}$, we first generate a prototype $W_{0}\in \mathbb {R}^{a\times b}$ using the following scenario. We assume three groups of columns with sizes {4,3,3} in *W*_0_, respectively. First, the input features are randomly selected for the three output groups (two for the first group, three for the second group, and three for the third group). We then randomly choose another feature, which is used for all the three groups, and further choose another feature for only the second and third groups. Hence, three features in total are chosen for the first group, five for the second group, and five for the third group, such that the spatial relationships between the three groups are different. We then generate *W* by putting its entries in (*i,j*)-th block *W*^*ij*^=*W*_0_(*i,j*)+*ε*, where *ε*∼*N*(0,0.1), in either a sparse or dense way. Two datasets, Data1.0 and Data2.0, are generated with the sparse and the relatively dense scenarios, respectively.

We then obtain the similarity among the groups of tasks by the Gaussian kernel calculated among the columns of *W*_0_. To show the effectiveness of using the super-graph structure, we randomly perturb *W*_0_ by changing *t* nonzero entries to zero and changing *t* zeros entries to nonzero, in different levels with *t*=2,5,10. With the same *A* and the perturbed *W*_0_s, we generate datasets Data1.1, Data1.2, Data1.3 and Data2.1, Data2.2, Data2.3 based on Data1.0 and Data2.0, respectively. Note that the first digit in the name of the dataset indicates whether the entries in the nonzero blocks of *W* is sparse or not, while the last digit represents the levels of the perturbation.

#### B. Evaluating the BBSS-MTL without super-graph structure

With the eight datasets, we first check the prediction performance of the proposed block sparse multi-task learning method BBSS-MTL without super-graph structure, i.e. *λ*_3_=0. We compare BBSS-MTL with other multi-task methods, including *ℓ*_1_ regularization and *ℓ*_2,1_ regularization. For each dataset, we first randomly split the *p*=50 samples into five folds. Four of the folds are taken as training data and the remaining fold is taken as test data, in turn. Parameters are chosen by cross-validation on the training data only. Once *W*^∗^ is calculated from the training data, the mean squared error on the test data ($\phantom {\dot {i}\!} {MSE}_{W^{*}} = \|B- {AW}^{*}\|_{F}$) is used to measure the performance of the learning.

Table 3 shows the mean and standard deviation of the MSEs calculated by 50 different split of the datasets. The results show that our BBSS-MTL method performs best for all of the datasets. For Data1.*, where each block of *W* has sparse entries, *ℓ*_1_ regularization is the second best and *ℓ*_2,1_ is the worst. For Data2.*, both *ℓ*_1_ and *ℓ*_2,1_ perform poorly, but our approach performs well.

**Table 3:** The mean squared error of different regularization methods

	*ℓ*_1_	*ℓ*_2,1_	BBSS-MTL(*λ*_1_,*λ*_3_=0)	BBSS-MTL(*λ*_3_=0)
Data1.0	3.472 ±0.023	4.272 ±0.055	**3.328** ±**0.018**	**3.325** ±**0.017**
Data1.1	3.401 ±0.020	4.324 ±0.058	**3.269** ±**0.016**	**3.268** ±**0.018**
Data1.2	3.409 ±0.019	4.238 ±0.072	**3.265** ±**0.016**	**3.266** ±**0.016**
Data1.3	3.513 ±0.019	4.430 ±0.076	**3.388** ±**0.019**	**3.391** ±**0.020**
Data2.0	1.313 ±0.031	1.315 ±0.018	**1.014** ±**0.007**	**1.016** ±**0.007**
Data2.1	1.331 ±0.037	1.337 ±0.025	**1.031** ±**0.007**	**1.029** ±**0.007**
Data2.2	1.372 ±0.028	1.383 ±0.026	**1.087** ±**0.008**	**1.089** ±**0.008**
Data2.3	1.506 ±0.025	1.529 ±0.030	**1.199** ±**0.011**	**1.200** ±**0.008**

Best results are shown in bold

**Fig. 2:**
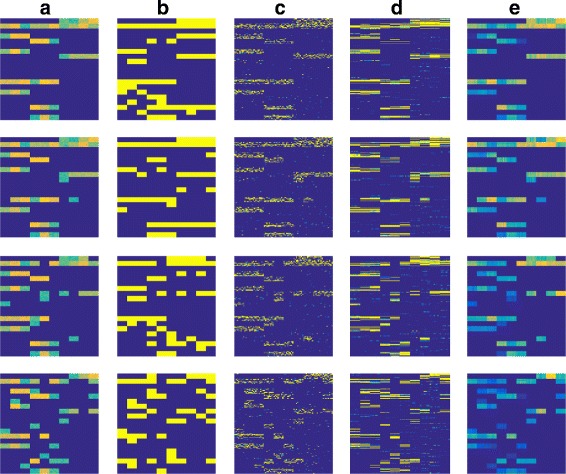
The calculated *W* of different methods for Data2.0 (1 ^st^ row), Data2.1 (2 ^nd^ row), Data2.2 (3 ^rd^ row) and Data2.3 (4^th^ row). Column (**a**): Ground truth *W*; (**b**): *W* connectivity mapping analysis test; (**c**): *W* *ℓ*_1_ regularization; (**d**): *ℓ*_2,1_ regularization; (**e**): BBSS-MTL with *λ*_3_=0. BBSS-MTL performs best among all the methods in all simulated data set for learning the *W*

In Fig. 2, we show the calculated *W* for Data2.0, Data2.1, Data2.2, and Data2.3, using a connectivity mapping analysis test (introduced in “Connectivity mapping analysis” section) and different types of regularization. The selected regularization parameters are chosen to be 0.005, 0.01 and 0.2 for *ℓ*_1_, *ℓ*_2,1_ and BBSS-MTL, respectively. We observe that both *ℓ*_1_ regularization and *ℓ*_2,1_ regularization have false positive discoveries, and that BBSS-MTL could enforce the non-interesting blocks in *W* to be exactly zero for all the four datasets.

#### C. Evaluating the BBSS-MTL with super-graph structure

To further evaluate the performance of our BBSS-MTL approach, we designed the following experiments. Suppose we have *A* and *B*, the latter of which is generated by a ground truth *W*: the nonzero blocks in *W* can be discovered by BBSS-MTL without super-graph structure. However, if the given *B* is generated by a perturbation $\tilde {W}$ of *W*, can we recover the useful information in *W* from *A* and *B*?

**Fig. 3:**
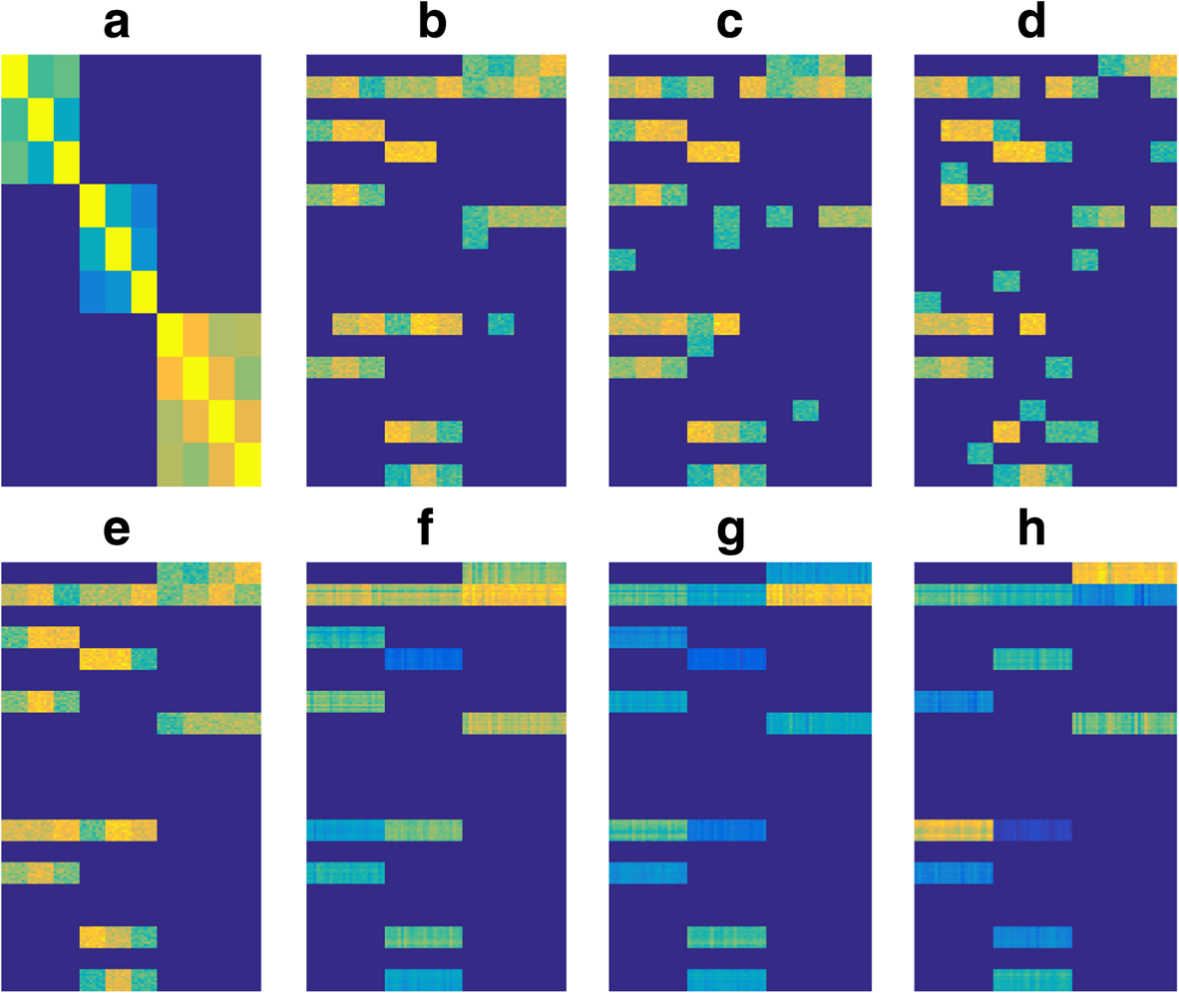
The simulation results for BBSS-MTL with super-graph structure. (**a**): The structure similarity matrix *K*; (**b**, **c**, **d**): The perturbed *W*s for Data2.1, Data2.2 and Data2.3; (**e**): The ground truth *W*; (**f**, **g**, **h**): The recovered *W* with *K* by BBSS-MTL. BBSS-MTL can recover the true *W* with the help of structure similarity, even when the datasets are perturbed

Note that Data2.1, Data2.2, and Data2.3 are generated based on the perturbations from the *W* in Data2.0. For this *W*, we also have the similarity matrix *K* among the task groups, which was computed as a Gaussian kernel among the columns of its prototype *W*_0_. With the side information *K*, we could apply our BBSS-MTL with super-group structure on the three datasets to determine whether the computed *W*^∗^ matches the ground truth *W*. The Fig. 3 shows the results, in which the nonzero blocks in the true *W* can be recovered by BBSS-MTL from all the three levels of perturbation.

### Connectivity mapping analysis

As a baseline to compare the effect of the compound treatment and the gene knock-down, our adopted method is based on the connectivity mapping analysis test [15], which is based on the Kolmogorov-Smirnov statistic [29]. Given a set of up- and down-regulated genes *S*_*up*_ and *S*_*dn*_ and a vector of differential gene expressions *E* with length *n*, where *S*_*up*_ and *S*_*dn*_ are subsets of *n* genes whose differential expression are measured in the given vector, the connectivity mapping analysis tests whether the given gene set is enriched in the given gene expression vector using the Kolmogorov-Smirnov test. Specifically, the analysis starts by ranking the expression vector *E* and then constructs a new vector *V* indicating the position of each gene. The up-connectivity score *CS*_*up*_ is then calculated from the following two values:

$$a = \max\limits_{i\in s_{up}}\left(\frac{i}{|s_{up}|}-\frac{V(i)}{n}\right), b = \max\limits_{i\in s_{up}}\left(\frac{V(i)}{|s_{up}|}-\frac{j-1}{|s_{up}|}\right). $$

Then *CS*_*up*_=*a* if *a*>*b* or *CS*_*up*_=−*b* if *b*>*a*. Similarly, we can calculate *CS*_*dn*_ for the down-regulated gene set. The connectivity score *CS*=0 if *CS*_*up*_ and *CS*_*dn*_ have the same sign; otherwise *CS*=*CS*_*up*_−*CS*_*dn*_. The null distribution is obtained by randomly permuting *E* 1000 times and then calculating the test statistic.

We then consider the case in which we compare two signature vectors of differential gene expressions: one from a drug treatment and the other one from a gene knock-down. We follow the method used in [30]. Firstly, we rank the two vectors to get the two up- and down-regulated gene sets. We then use the connectivity mapping analysis test twice to test whether the up- and down-regulated gene sets from one vector are enriched in the other ones. Finally, the p-values are summarized using the Fisher inverse chi-square test statistic. The null distribution is also obtained by random permutations.

As mentioned, the above-described method cannot be directly applied to compare a drug treatment and a gene knock-down, as the drug treatment and gene knock-down are represented by groups of signatures in L1000 dataset. Therefore, we have compared all the pairs of signatures from a drug treatment and a gene knock-down and chosen the smallest p-value. Finally, we performed one-to-one mapping on all the pairs of drug treatments and gene knock-downs and ranked them using the *p*-values.

### Experimental results

For each of the induced real datasets, we applied our BBSS-MTL approach to find the associations among the drugs and the knock-down genes. For the parameters, we set *λ*_1_=1 and *λ*_3_=1*e*+4. We chose the parameter set *Λ*_2_ such that the smallest one could get dense *W* and the largest one could get *W* with all zeros. With these parameter setting, stability analysis was used to obtain a stability score for each drug-gene pair.

Since the approved drug-target pairs are limited, we evaluated our predicted potential drug targets by integrating the gene function information in the Gene Ontology or the protein-protein interactions in the PPI network. We define potential drug targets of a drug as those genes that are down- or upstream and closely related to the real targets in pathways. We first computed the semantic scores among all the genes in each of our five datasets using the R software package Gosim [31]. Two genes with a high semantic score are considered to have similar gene functions. If a predicted gene’s semantic similarity score with any known target gene of a drug is higher than 0.8, it is considered as a potential target for the drug. By comparing our results with the GO-based scores, we could calculate the AUC (area under curve) values AUC_GO for each cell line dataset. We also evaluated our results by using a human PPI network, Human Protein Reference Database (HPRD) [22]. We computed pairwise distances among all genes in each of our induced datasets by their shortest paths. Two genes with a connection on the PPI network are considered to have physical interactions with each other. Thus, if a predicted gene is connected to any known target gene of a drug, it is considered as a potential target for a drug. We computed AUC_PPI by comparing our results with the PPI-based scores.

Table 4 shows the AUC values using Lasso, connectivity mapping and our BBSS-MTL for the five cell line datasets. For Lasso, we simply average the expression levels of different treatments (different knock-downs) for each drug (gene), and apply single task lasso to calculate association stability scores similar to the BBSS-MTL approach. We can see that for the small dataset of cell line SW480, our method could achieve the highest AUC values among all the five datasets, based on either GO information or PPI information. The results obtained for the other four cell lines are slightly lower. For almost all the datasets, our approach performs significantly better than the connectivity mapping approach, which attempts to find the associations by one-to-one mapping. Our approaches obtained higher AUC values based on either GO information or PPI information, which may suggest that the multi-task learning could discover useful multiple targets that are affected by the same drugs.

**Table 4:** The area under the ROC curve (AUC) for the prediction

Cell line	Lasso		Connectivity mapping		BBSS-MTL	
	AUC_GO	AUC_PPI	AUC_GO	AUC_PPI	AUC_GO	AUC_PPI
HCC515	0.528	0.510	0.456	**0.549**	**0.592**	0.537
HT29	0.458	0.563	0.479	0.556	**0.558**	**0.603**
PC3	0.491	0.503	0.509	0.561	**0.550**	**0.580**
SW480	0.500	0.400	0.444	0.609	**0.769**	**0.719**
MCF7	0.541	0.588	0.492	0.541	**0.571**	**0.606**

## Discussion

### Additive effects of NFKB1 and 1KBKB for immunologic drugs

We first investigated the results obtained from the smallest dataset generated from the SW480 cell line. Table 5 shows a list of potential targets for the drugs discovered from the SW480 cell lines. The SW480 dataset only includes four drugs and six genes. The four drugs are Thalidomide, Valproic acid, Sirolimus and Auranofin and the six genes are IKBKB, NFKB1, MTOR, HDAC2, PPARG and AR. Among these genes and drugs, NFKB1 is an approved target for Thalidomide, and IKBKB is an approved target for Auranofin. The results show that these known targets for the two drugs are discovered (in rows 3 and 7 of the table, respectively), and NFKB1 and IKBKB could both have additive effects on the drug treatment of Thalidomide or Auranofin. This is highly likely because NFKB1 and IKBKB are close in the NF-Kappa-B signaling pathway, which is a critical pathway for immune response. NFKB1 forms the NF-kappa-B complex and is known to be inhibited by I-kappa-B proteins, while IKBKB is a kinase which phosphorylates serine residues on the I-kappa-B proteins and further activates the NF-kappa-B complex. Our approach successfully recovers the connection between these two genes. The discovered link between the drug Auranofin and the gene NFKB1 could be further supported by studies on Auranofin [32]. In addition, Sirolimus is also known to be also highly related to the immune system. Also, studies have shown that Valproic acid can induce neural tube defects, which is related with maternal immune system [33, 34]. Therefore, NFKB1 and IKBKB may also have additive effects for the treatments with immunologic agents. The top eight predicted pairs show strong connections between the two above-mentioned genes and all four immunologic drugs.

**Table 5:** Top predictions of potential drug targets on SW480 cell line

Drug	Gene	Stability score
Thalidomide	IKBKB	0.927
Valproic acid	IKBKB	0.920
Thalidomide	NFKB1	0.913
Valproic acid	NFKB1	0.900
Sirolimus	IKBKB	0.880
Sirolimus	NFKB1	0.873
Auranofin	IKBKB	0.847
Auranofin	NFKB1	0.847

### A unified bipartite graph of drugs and targets in three cell lines

**Fig. 4:**
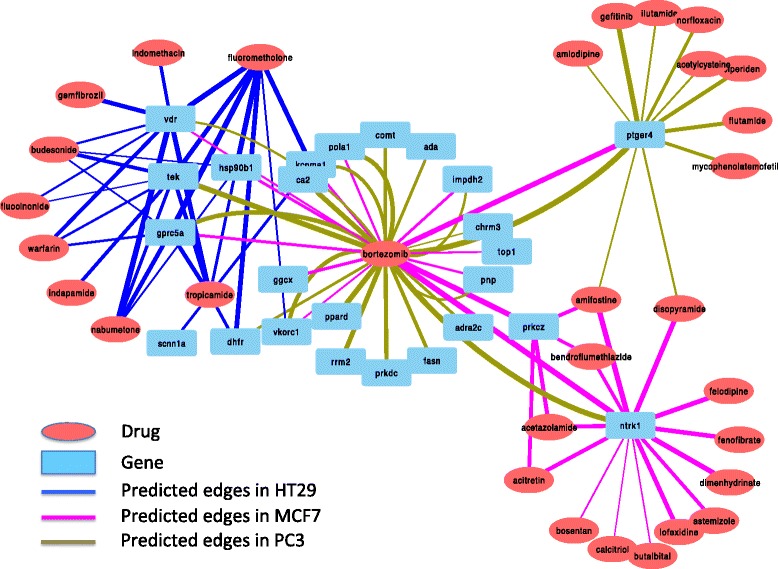
The unified bipartite graph by BBSS-MTL across the three cell line datasets of HT29, MFC7 and PC3

Figure 4 shows our findings across the three cell line datasets of HT29, MFC7 and PC3. The top 30 associations between drugs and genes detected by the proposed method BBSS-MTL on the datasets from the three cell lines are depicted in a bipartite graph of drugs and genes. Interestingly, all the drugs and genes in the three datasets are connected in a unified bipartite graph, and co-modules of drugs and genes could be observed. There are some novel findings from the unified bipartite graph. There are roughly five co-modules of drugs and targets in the graph, including the co-module of the drugs Fluorometholone, Tropicamide, Budesonide, Indapamide, Nabumetone, Warfarin and Fluocinonide and the genes VDR and TEK, the co-module of Bortezomib and its several potential targets CA1, ADA,FASN PRKDC,RRM2, PPARD, COMT,GGCX, PRKCZ, the co-module of PTGER4 and several drugs of Amlodipine, Gefitinib, Nilutamide, Norfloxacin, Acetylcysteine, Biperiden, Flutamide, Mycophenolatemofetil, Amifostine, Disopyramide and Bortezomib, the co-module of the gene NTRK1 and drugs Amifostine, Disopyramide, Bortezomib, Felodipine, Fenofibrate, Dimenhydrinate, Astemizole, Lofexidine, Acitretin, Acetazolamide, Bendroflumethiazide, Amifostine and Disopyramide, the co-module of the gene PRKCZ and the drugs Bortezomib, Amifostine, Acitretin, Acetazolamide and Bendroflumethiazide.

Some of the novel findings can be supported by other references or pathway analysis. For example, using the dateset of HT29 cell line, a group of drugs Fluorometholone, Tropicamide, Budesonide, Indapamide, Nabumetone, Warfarin and Fluocinonide are predicted to be related with a group of genes VDR and TEK. The gene VDR is a vitamin D receptor in lipid metabolism and calcium reabsorption. In the presence of a ligand, VDR binds to vitamin D response elements to either increase or repress transcription of target genes. Two known targets of Nabumetone - PTGS1 and PTGS2 - are also known to be in the pathway of lipid metabolism. Nabumetone is used for the treatment of osteoarthritis and rheumatoid arthritis. McAlindon et al. [35] showed that vitamin D may prevent progression of osteoarthritis, and Athanassiou et al. [36] pointed out that reduced vitamin D intake has been linked to increased susceptibility to the development of rheumatoid arthritis. This implies that knocking down VDR might have similar effects to taking Nabumetone, and that VDR could be a potential target for Nabumetone. The known targets of Tropicamide - CHRM1, CHRM2, CHRM3 and CHRM4 - are known to be in the pathway of calcium signaling pathway, which is likely to be related to VDR. The primary target of the drug Fluorometholone is the glucocorticoid receptor (GR). The bound receptor-ligand complex further binds to many glucocorticoid response elements (GREs) in the promoter region of the target genes. The VDR gene contains a number of putative GREs, and it has been proven that VDR could regulate the expression of glucocorticoid [37]. Therefore, the gene VDR could have an additive effect for the treatment of Fluorometholone. The gene TEK, a receptor tyrosine kinase, is also likely to have an effect on this drug. Kuo et al. [38] showed that many glucocorticoid-regulated genes affect receptor tyrosine kinase signalling. GR primary targets could inhibit the insulin/IGF1 pathway, which propagates through receptor tyrosine kinases. Knocking down TEK may modulate the activity of this pathway and further regulate the expression of GR. There is little evidence that VDR is close to TEK in the same pathway, and our results suggest that the two genes might be affected together in different pathways by the five drugs, including Fluorometholone and Nabumetone.

It is also interesting that we found edges between drugs and genes in multiple cell lines by BBSS-MTL. For example, we found that genes NTRK1, PTGER4, POLA1, VKORC1, GPRC5a, VDR, KCNMA1, IMPDH2 and PNP could be potential targets of the drug Bortezomib in both cell lines of MCF7 and PC3. The stability score of Bortezomib and NTRK1 is ranked first in the MCF7 cell line and second in the PC3 cell line by BBSS-MTL. Bortezomib is known to inhibit the 26S proteasome, which modulates the activity in the division of multiple myeloma and leukemic cells, and further induces apoptosis. The gene NTRK1 also modulates the activity of the apoptosis pathway. Therefore, it is likely that NTRK1 is the potential target of the drug Bortezomib or close to its target in the downstream pathways. We note that TEK is also predicted to be related to the drug Bortezomib, in the cell line of PC3, which means that the results from the two cell lines connect NTRK1 and TEK through Bortezomib. In fact, the genes NTRK1 and TEK are both receptor tyrosine kinases and it is known that 26S proteasome can degrade receptor tyrosine kinases [39]. The epidermal growth factor receptor (EGFR) is a known target for the drug Gefitinib. From the PC3 cell line, we could recover the connection between Gefitinib and the gene PGE4. It is known that the association of PGE4 with *β*-arrestin 1 and c-Src signaling complex could result in the transactivation of EGFR [40], which shows the high probability that PGE4 could take effect with EGFR together for the treatment of the drug Gefitinib.

To summarize, the results across different cell lines in whole graph presented in Fig. 4 show plenty of interesting and novel findings, including the unified connected bipartite graph, the co-modules of drugs and genes in the graph, the duplicate edges across multiple cell lines, and meaningful connections of genes.

## Conclusion

In this paper, we have proposed a bipartite block-wise sparse multi-task learning approach BBSS-MTL for discovering multiple targets for drugs using the LINCS L1000 dataset. We assume that the effect of a drug treatment can be approximated by adding the effects of all its single-target knock-downs and considering additive effects of multiple targets for a drug treatment. Our results show that our model could achieve higher accuracy in detecting potential drug targets than the widely used but simpler pairwise connectivity mapping. Interesting and novel discoveries by our methods, such as new biologically meaningful drug target candidates, the modules of the drugs and genes from the three different cell lines, and the duplicate edges predicted from different cell lines, also reflect the effectiveness of our approaches.

However, there are some limitations of our proposed approaches. For example, the agonistic effect cannot be reflected from the knock-down experiments. A drug generally only inhibits part of the protein functions, while knocking down a gene may reduce all its functions. Besides, our additivity assumption is a simplification of the complexity of the underlying biological system. Still, its better performance implies that it describes the biological system in the L1000 problem much better than the widely used pairwise mapping. It might be possible to get rid of the additivity assumption by constructing a more complicated nonlinear model that considers the interactions among multiple targets of a drug treatment. However, this will lead to a much higher computational load, which currently renders genome-wide analyses infeasible. This problem will be a topic of future work.

## References

[1] Lu JJ (2012). Multi-target drugs: The trend of drug research and development. PLoS ONE.

[2] Reddy AS, Zhang S (2013). Polypharmacology: drug discovery for the future. Expert Rev Clin Pharmacol.

[3] Paolini GV (2006). Global mapping of pharmacological space. Nat Biotech.

[4] Csermely P (2005). The efficiency of multi-target drugs: the network approach might help drug design. Trends Pharmacol Sci.

[5] Koutsoukas A (2011). From in silico target prediction to multi-target drug design: Current databases, methods and applications. J Proteome.

[6] Lounkine E (2012). Large-scale prediction and testing of drug activity on side-effect targets. Nature.

[7] Martin YC (2002). Do structurally similar molecules have similar biological activity?. J Med Chem.

[8] Csermely P (2013). Structure and dynamics of molecular networks: A novel paradigm of drug discovery: A comprehensive review. Pharmacol Ther.

[9] Li L (2010). Predicting enzyme targets for cancer drugs by profiling human metabolic reactions in nci-60 cell lines. BMC Bioinformatics.

[10] Li L (2014). Mpgraph: multi-view penalised graph clustering for predicting drugtarget interactions. IET Syst Biol.

[11] Isik Z (2015). Drug target prioritization by perturbed gene expression and network information. Sci Rep.

[12] Laenen G (2013). Finding the targets of a drug by integration of gene expression data with a protein interaction network. Mol BioSyst.

[13] Liu C (2015). Compound signature detection on lincs l1000 big data. Mol BioSyst.

[14] Subramanian A (2016). A next generation connectivity map: L1000 platform and the first 1,000,000 profiles. Cell.

[15] Lamb J (2006). The connectivity map: Using gene-expression signatures to connect small molecules, genes, and disease. Science.

[16] Subramanian A (2005). Gene set enrichment analysis: a knowledge-based approach for interpreting genome-wide expression profiles. PNAS.

[17] Additive roles of XPA and MSH2 genes in uvb-induced skin tumorigenesis in mice. DNA Repair. 2002; 1(11):935–40. 10.1016/s1568-7864(02)00144-1 12531021

[18] Alper H, Miyaoku K, Stephanopoulos G (2005). Construction of lycopene-overproducing e. coli strains by combining systematic and combinatorial gene knockout targets. Nat Biotechnol.

[19] Phillips PC (2008). Epistasis — the essential role of gene interactions in the structure and evolution of genetic systems. Nat Rev Genet.

[20] Wishart DS (2006). Drugbank: a comprehensive resource for in silico drug discovery and exploration. Nucleic Acids Res.

[21] Ashburner M (2000). Gene ontology: tool for the unification of biology. Nat Genet.

[22] Keshava Prasad TS, etal (2009). Human protein reference database—2009 update. Nucleic Acids Res.

[23] Kanehisa M, Goto S (2000). Kegg: Kyoto encyclopedia of genes and genomes. Nucleic Acids Res.

[24] Prabhakara S, Acharya R (2010). Simcomp: A hybrid soft clustering of metagenome reads. PRIB’10.

[25] Swirszcz G, Lozano AC (2012). Multi-level lasso for sparse multi-task regression. ICML.

[26] Zhou J (2012). Modeling disease progression via fused sparse group lasso. SIGKDD.

[27] Goncalves AR (2014). Multi-task sparse structure learning. CIKM.

[28] Chen X, et al. Graph-structured multi-task regression and an efficient optimization method for general fused lasso. 2010. arXiv:1005.3579v1.

[29] Hollander M, Wolfe DA (1999). Nonparametric Statistical Methods. Wiley series in probability and statistics.

[30] Hoshida Y, Brunet J-P, Tamayo P, Golub TR, Mesirov JP (2007). Subclass mapping: Identifying common subtypes in independent disease data sets. PLoS ONE.

[31] Yu G (2010). Gosemsim: an r package for measuring semantic similarity among go terms and gene products. Bioinformatics.

[32] Jeon K-I (2003). Gold compound auranofin inhibits ikappab kinase (ikk) by modifying cys-179 of ikkbeta subunit. Exp Mol Med.

[33] Bennett G (2000). Valproic acid-induced alterations in growth and neurotrophic factor. Reprod Toxicol.

[34] Deeb SA (2000). Vitamin e decreases valproic acid induced neural tube defects in mice. Neurosci Lett.

[35] McAlindon TE (1996). Relation of dietary intake and serum levels of vitamin d to progression of osteoarthritis of the knee among participants in the framingham study. Ann Intern Med.

[36] Kostoglou-Athanassiou A (2012). Vitamin d and rheumatoid arthritis. Ther Adv Endocrinol Metab.

[37] Hidalgo AA (2010). Glucocorticoid regulation of the vitamin d receptor. J Steroid Biochem Mol Biol.

[38] Kuo T (2012). Genome-wide analysis of glucocorticoid receptor-binding sites in myotubes identifies gene networks modulating insulin signaling. PNAS.

[39] Sepp-Lorenzino L (1995). Herbimycin a induces the 20 s proteasome- and ubiquitin-dependent degradation of receptor tyrosine kinases. J Biol Chem.

[40] Buchanan FG (2006). Role of *β*-arrestin 1 in the metastatic progression of colorectal cancer. PNAS.

